# Serum klotho as a novel biomarker for metabolic syndrome: findings from a large national cohort

**DOI:** 10.3389/fendo.2024.1295927

**Published:** 2024-03-04

**Authors:** Li Yuguang, Yu Chang, Naifei Chen, Yixin Zhao, Xinwei Zhang, Wei Song, Jin Lu, Xiangliang Liu

**Affiliations:** ^1^Cancer Center, The First Hospital of Jilin University, Changchun, China; ^2^Department of Gastroenterology,The First Hospital of Jilin University, Changchun, China

**Keywords:** metabolic syndrome, klotho, mortality, biomarkers, NHANES

## Abstract

**Background:**

Metabolic syndrome is a cluster of metabolic abnormalities that significantly increase the risk of cardiovascular disease and mortality. The identification of novel biomarkers associated with mortality in patients with metabolic syndrome could facilitate early risk stratification and targeted interventions.

**Methods:**

We conducted a large prospective cohort study using data from five cycles (2009-2016) of the National Health and Nutrition Examination Survey (NHANES) database, including a total of 40,439 participants. Logistic regression analysis was used to assess the association between serum klotho protein levels and metabolic syndrome, while Cox regression analysis was employed to examine the correlation between serum klotho levels and all-cause mortality. Mortality data were updated until December 31, 2019.

**Results:**

After adjusting for demographic and socioeconomic confounders, the logistic regression model demonstrated that higher serum klotho levels were significantly associated with a decreased prevalence of metabolic syndrome (OR [95% CI] Highest vs. lowest quartile: 0.84 [0.70-0.99], P=0.038). In the Cox regression model, elevated klotho levels were found to significantly reduce the risk of all-cause mortality among individuals with metabolic syndrome (HR [95% CI] Highest vs. lowest quartile: 0.68 [0.51-0.90], P=0.006).

**Conclusion:**

Serum klotho levels were found to be inversely associated with the prevalence of metabolic syndrome, independent of potential confounding factors such as demographics, socioeconomic status, and lifestyle factors. Furthermore, higher klotho levels strongly indicated a lower risk of all-cause mortality in individuals with metabolic syndrome.

## 1 Introduction

Metabolic syndrome refers to a cluster of metabolic abnormalities and cardiovascular risk factors, including abdominal obesity, dyslipidemia, hypertension, and hyperglycemia (1). The prevalence of metabolic syndrome has increased substantially worldwide over the past few decades, imposing a huge burden on public health (2). Epidemiological studies have consistently shown that metabolic syndrome significantly increases the risk of developing cardiovascular disease, type 2 diabetes, chronic kidney disease, and other metabolic disorders (3–5). Moreover, metabolic syndrome is associated with elevated risks of all-cause and cardiovascular mortality (6, 7). Given the high prevalence and detrimental health consequences of metabolic syndrome, identifying novel risk factors and prognostic biomarkers is of great importance to facilitate early prevention and treatment.

Klotho is an anti-aging protein predominantly produced in the kidney, brain, and endothelial cells (8). A growing body of evidence suggests that Klotho is involved in the regulation of various physiological processes, including oxidative stress, inflammation, insulin sensitivity, glucose homeostasis, etc (3). Animal studies demonstrate that Klotho deficiency results in vascular calcification, accelerated atherosclerosis, and hypertension (9). In humans, higher Klotho levels are associated with reduced risks of chronic kidney diseases (CKD), cardiovascular diseases (CVD), and mortality (7, 10, 11). Preventing the reduction of Klotho levels and enhancing its production can reduce renal fibrosis, slow down the progression of CKD, and improve mineral metabolism in CKD patients. In light of the potential role of Klotho in metabolism-related diseases, recent studies begin to investigate the relationship between Klotho and metabolic syndrome, but the results remain inconsistent, especially regarding the prognostic value of Klotho (12–14).

In the present study, we aim to systematically assess the associations of serum Klotho levels with the prevalence and mortality of metabolic syndrome using a large nationally representative cohort. Our findings may provide novel insights into the pathogenic role of Klotho in metabolic syndrome and support Klotho as an independent prognostic biomarker.

## 2 Methods

### 2.1 Study population

**Figure 1:**
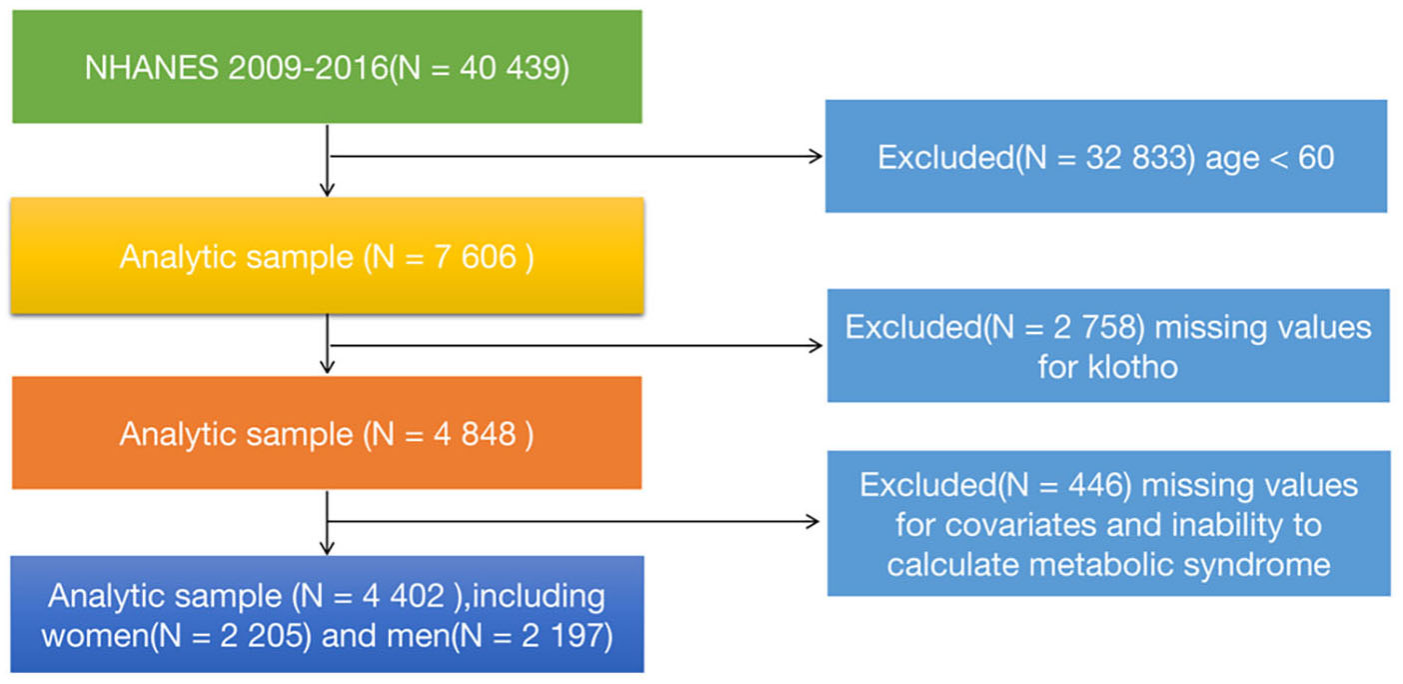
The flowchart of study and excluded participants.

The NHANES (National Health and Nutrition Examination Survey) database is a cross-sectional study approved by the Centers for Disease Control and Prevention (CDC) and the Institutional Review Board (IRB), aiming to assess the nutritional status and health of the US population. It utilizes a probability and stratified, multi-stage random sampling method. Participants undergo a series of questionnaire surveys, physical examinations, as well as blood and urine sample collection. For more specific details about the database, please refer to the official website (https://www.cdc.gov/nchs/NHANES/index.htm). We downloaded four consecutive cycles of NHANES data (2009-2010, 2011-2012, 2013-2014, 2015-2016) from the website. A total of 40,439 participants were initially included. We excluded participants aged <60 years (n=32,833), those with missing klotho data (n=2,758), those with missing covariate data or inability to determine the presence of metabolic syndrome (n=446). Finally, 4,402 participants (2,205 females and 2,197 males) were included in the analysis. More specific details can be seen in **Figure 1**.

### 2.2 Acquisition of klotho samples

The blood samples of individuals aged 60 and above from four cycles (2009-2016) were collected by the personnel of the NHANES study. The samples were stored in dry ice at approximately -80°C and sent to the Northwest Lipid Metabolism and Diabetes Research Laboratory at the University of Washington for analysis. Serum levels of Klotho were determined for fresh frozen samples using the principle of enzyme-linked immunosorbent assay (ELISA) with the human α-Klotho test kit (intra-assay coefficient of variation <3.6%, inter-assay coefficient of variation <11.4%, detection sensitivity: 6pg/mL). Each sample was analyzed in duplicate by trained analysts, and the average of the two values was calculated as the final result. Each sample was analyzed twice, and the analysis results were automatically transferred to the laboratory’s Oracle management system for evaluation by professional supervisors. If the repeated results exceeded 10%, the analysis was deemed invalid and a repeat sample analysis was required. For more detailed information on the Klotho measurement method, please refer to the NHANES website (15).

### 2.3 Diagnosis of metabolic syndrome

We screened participants diagnosed with metabolic syndrome based on guidelines provided by organizations such as the International Diabetes Federation (IDF) and the American Heart Association (AHA). The diagnosed individuals were required to meet three or more of the following criteria: 1) Male waist circumference ≥ 40 inches (102 cm), female waist circumference ≥ 35 inches (88 cm); 2) Elevated blood pressure: systolic pressure ≥ 130 mmHg or diastolic pressure ≥ 85 mmHg, or currently taking antihypertensive medication; 3) Abnormal blood lipid levels: triglycerides ≥ 150 mg/dL (1.7 mmol/L) or HDL < 40 mg/dL (1.03 mmol/L), or currently taking lipid-lowering medication; 4) Impaired glucose metabolism: fasting blood glucose ≥ 100 mg/dL (5.6 mmol/L) or currently using antidiabetic medication for blood sugar control (16).

### 2.4 Covariates

Covariates such as gender, poverty-income ratio (PIR), race, educational level, and smoking status were included in the analysis. PIR was categorized into three levels: ≤1, 1–3, and >3. Race was classified as Mexican American, Non-Hispanic Black, Non-Hispanic White, Other Hispanic, and other races (including multi-racial). Educational level was divided into three categories: below high school, high school, and college or above. Smoking status was grouped into three categories: former (having smoked more than 100 cigarettes in a lifetime but currently not smoking), never (having smoked fewer than 100 cigarettes in a lifetime), and now (having smoked more than 100 cigarettes in a lifetime and currently smoking all or some days).

### 2.5 Mortality

We linked the NHANES database with the National Death Index (NDI) database, which includes follow-up mortality statistics for participants in the NHANES survey through December 31, 2019. The NDI utilizes the International Classification of Diseases, 10th Edition (ICD-10), to determine the cause of death. Causes of death were categorized into six major groups: cardiovascular disease, Alzheimer’s disease, cerebrovascular disease, malignant neoplasms (cancer), chronic lower respiratory diseases, and other causes of death.

### 2.6 Statistical analysis

Continuous variables following a normal distribution were described using the mean (standard deviation, SD), and statistical differences were assessed using t-tests or analysis of variance (ANOVA). Non-normally distributed continuous variables were described using the median (interquartile range, IQR), and non-parametric tests were used to assess statistical differences. Categorical variables were described using frequencies (percentages), and chi-square tests were employed to determine statistical differences.

In this study, due to the skewed distribution of klotho data, we performed a log2 transformation to present the analysis results more accurately. Subsequently, we classified the concentration of klotho based on quartiles, with the first quartile set as the reference group. We established a multivariable logistic regression model to explore the correlation between serum klotho levels and metabolic syndrome, adjusted for confounding factors including gender, race, education level, PIR, and smoking status, presenting the results as odds ratios (ORs) with 95% confidence intervals (CIs).

We also plotted restricted cubic spline (RCS) graphs and employed ten-fold cross-validation to examine whether there was a nonlinear relationship between log2-transformed serum klotho levels and metabolic syndrome. Additionally, we constructed a multivariable Cox regression model and generated Kaplan-Meier survival curves to investigate the association between log2-transformed serum klotho levels in patients with metabolic syndrome and all-cause mortality. Restricted cubic splines were used, and likelihood ratio tests were conducted to assess the nonlinear relationship between log2-transformed serum klotho levels and all-cause mortality. To ensure robustness of the research findings, sensitivity analyses were performed by excluding patients with a death time <30 months to eliminate potential causal associations. All statistical analyses and data visualizations were performed by R 4.2.2.

## 3 Results

### 3.1 Baseline features

According to the presence of metabolic syndrome, a total of 4,402 participants aged 60 and above were included in this analysis. Among them, there were 2,197 male participants (49.9%) and 2,205 female participants (50.1%). Among these participants, 2,185 participants (49.6%) had metabolic syndrome, and this group was more likely to be female, have an income-to-poverty ratio ≥1, be non-Hispanic white, have a high school or higher education level, and be never smokers. For more detailed baseline information, please refer to **Table 1**.

**Table 1:** Baseline characteristic of the study population by Metabolic syndrome.

	Level	No metabolic syndrome	Metabolic syndrome	P-value
N (%)		2217 (50.36)	2185 (49.64)	
Klotho (%)				0.0934
	Q1	519 (23.41)	582 (26.64)	
	Q2	571 (25.76)	530 (24.26)	
	Q3	568 (25.62)	531 (24.30)	
	Q4	559 (25.21)	542 (24.81)	
Sex (%)				<0.0001
	Female	1026 (46.28)	1179 (53.96)	
	Male	1191 (53.72)	1006 (46.04)	
Poverty-income ratio (%)				<0.0001
	<1	378 (17.05)	481 (22.01)	
	[1, 3)	935 (42.17)	983 (44.99)	
	≥3	904 (40.78)	721 (33.00)	
Ethnicity (%)				<0.0001
	Mexican American	269 (12.13)	362 (16.57)	
	Non-Hispanic Black	491 (22.15)	400 (18.31)	
	Non-Hispanic White	989 (44.61)	975 (44.62)	
	Other Hispanic	237 (10.69)	270 (12.36)	
	Other Race - Including Multi-Racial	231 (10.42)	178 (8.15)	
Education (%)				<0.0001
	Low high school	583 (26.30)	699 (31.99)	
	High school	465 (20.97)	518 (23.71)	
	College or above	1169 (52.73)	968 (44.30)	
Smoke (%)				0.0313
	Former	792 (35.72)	864 (39.54)	
	Never	1090 (49.17)	1004 (45.95)	
	Now	335 (15.11)	317 (14.51)	
Calcium (mean (SD))		2.357 (0.093)	2.361 (0.099)	0.1812
Phosphorus (mean (SD))		1.200 (0.093)	1.198 (0.186)	0.7715

### 3.2 The association between klotho and metabolic syndrome

**Figure 2:**
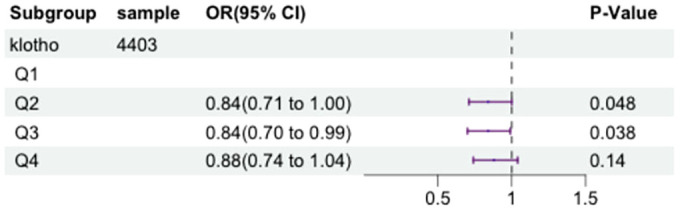
The forest plot displaying the associations between the levels of klotho and metabolic syndrome.

To further investigate the association between serum klotho levels and the occurrence of metabolic syndrome, we constructed a logistic regression model adjusting for above confounding factors. The serum klotho data was log2-transformed, and the logarithmically transformed concentrations were categorized according to quartiles (Q1, Q2, Q3, Q4). Compared to the first quartile of klotho, the Q2 group showed 16% decrease in metabolic syndrome risk (OR=0.84,95% CI = 0.71-1.00, P = 0.048), and the Q3 group showed 16% decrease in metabolic syndrome risk (OR=0.84,95% CI = 0.70-0.99, P = 0.038). Please refer to **Figure 2** for specific details.

**Figure 3:**
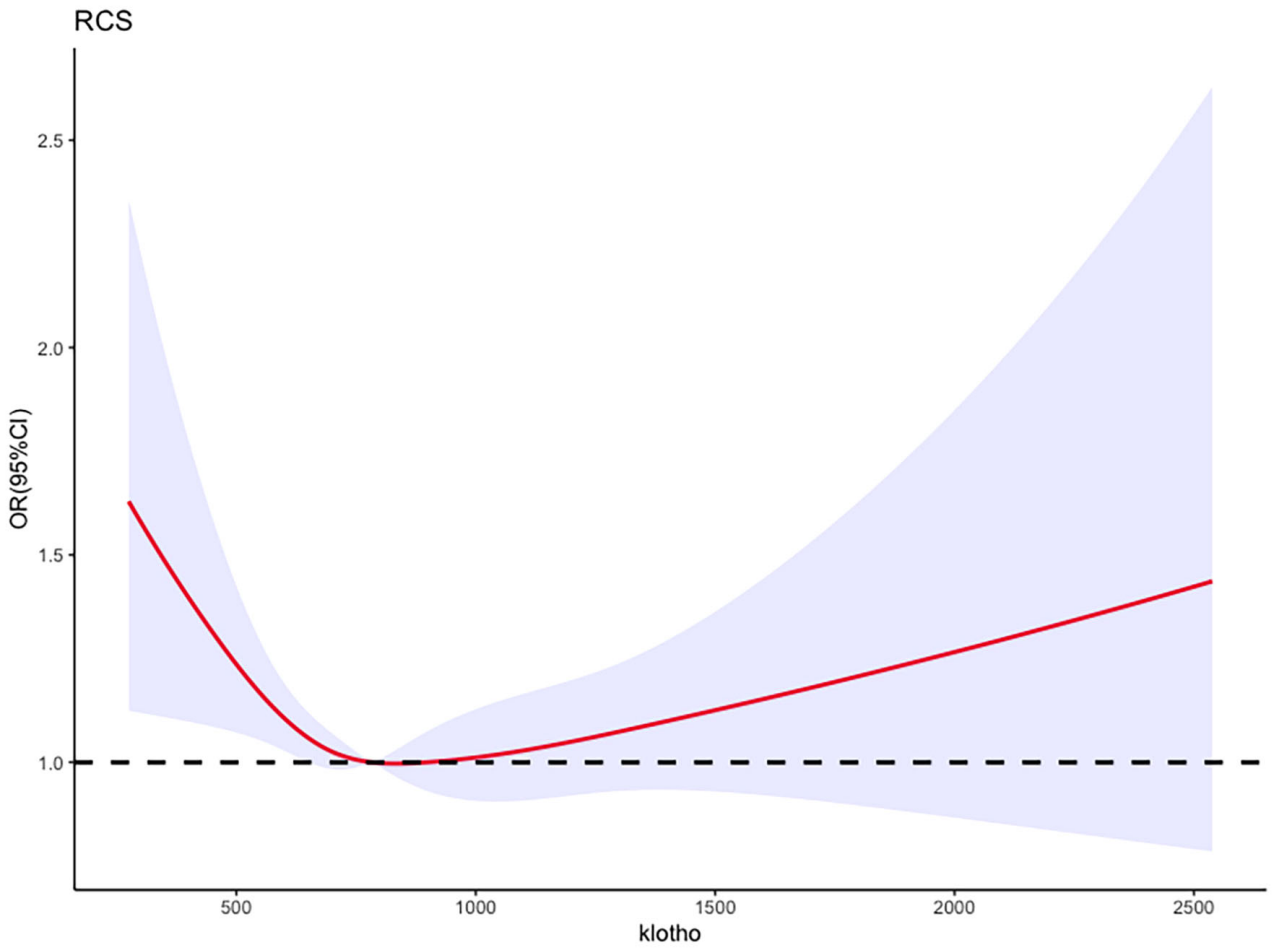
Adjusted restricted cubic spline curve between the levels of klotho and metabolic syndrome.

In the logistic regression model, with adjustment for above confounding factors, **Figure 3** depicts a RCS plot describing the dose-response relationship between log2-transformed serum klotho levels and the risk of metabolic syndrome. Using the first quartile of klotho as the reference value, a significant nonlinear correlation was observed between serum klotho levels and the risk of metabolic syndrome (the P value for non-linearity was 0.0152).

### 3.3 Association between serum klotho levels and risk of mortality in patients with metabolic syndrome

**Figure 4:**
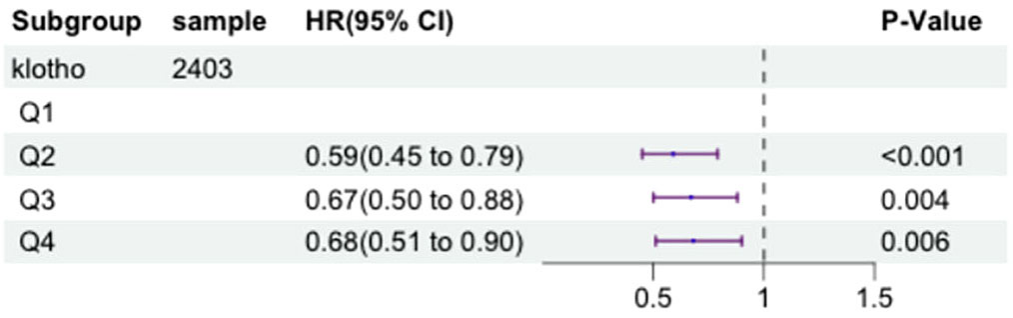
The forest plot displaying Hazard Ratios of klotho levels and all-cause mortality in patients with metabolic syndrome.

We selected a total of 2185 participants with metabolic syndrome for our study. Serum klotho levels were transformed logarithmically and divided into quartiles. To investigate the relationship between serum klotho levels and the risk of mortality in patients with metabolic syndrome, we employed a multivariate Cox proportional hazards model, adjusting for above confounding factors. **Figure 4** illustrates the results of this analysis.

**Figure 5:**
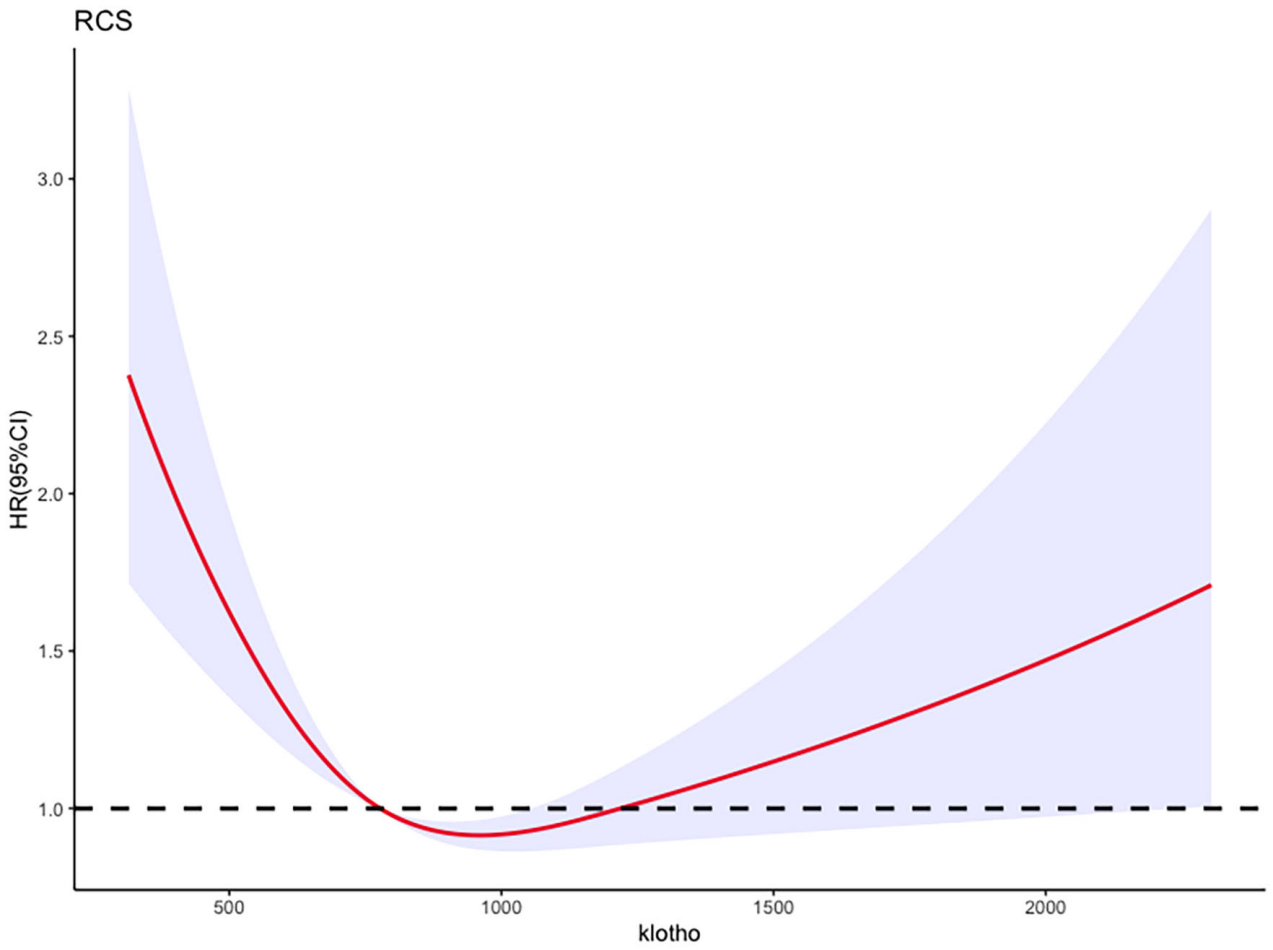
Adjusted restricted cubic spline curve between the levels of klotho and all-cause mortality in patients with metabolic syndrome.

After adjusting for these factors, compared to the first quartile (Q1) of log2-transformed serum klotho, the second quartile (Q2) showed a 41% decrease in all-cause mortality risk (HR=0.59, 95% CI=0.45-0.79, P<0.001), the third quartile (Q3) showed a 33% decrease in all-cause mortality risk (HR=0.67, 95% CI=0.50-0.88, P=0.004), and the fourth quartile (Q4) showed a 32% decrease in all-cause mortality risk (HR=0.68, 95% CI=0.51-0.90, P=0.006). These results indicate a significant association between lower all-cause mortality risk in patients with metabolic syndrome and higher log2-transformed serum klotho levels in the Q2, Q3, and Q4 groups compared to the Q1 group. More details can be seen in **Figure 5**.

**Figure 6:**
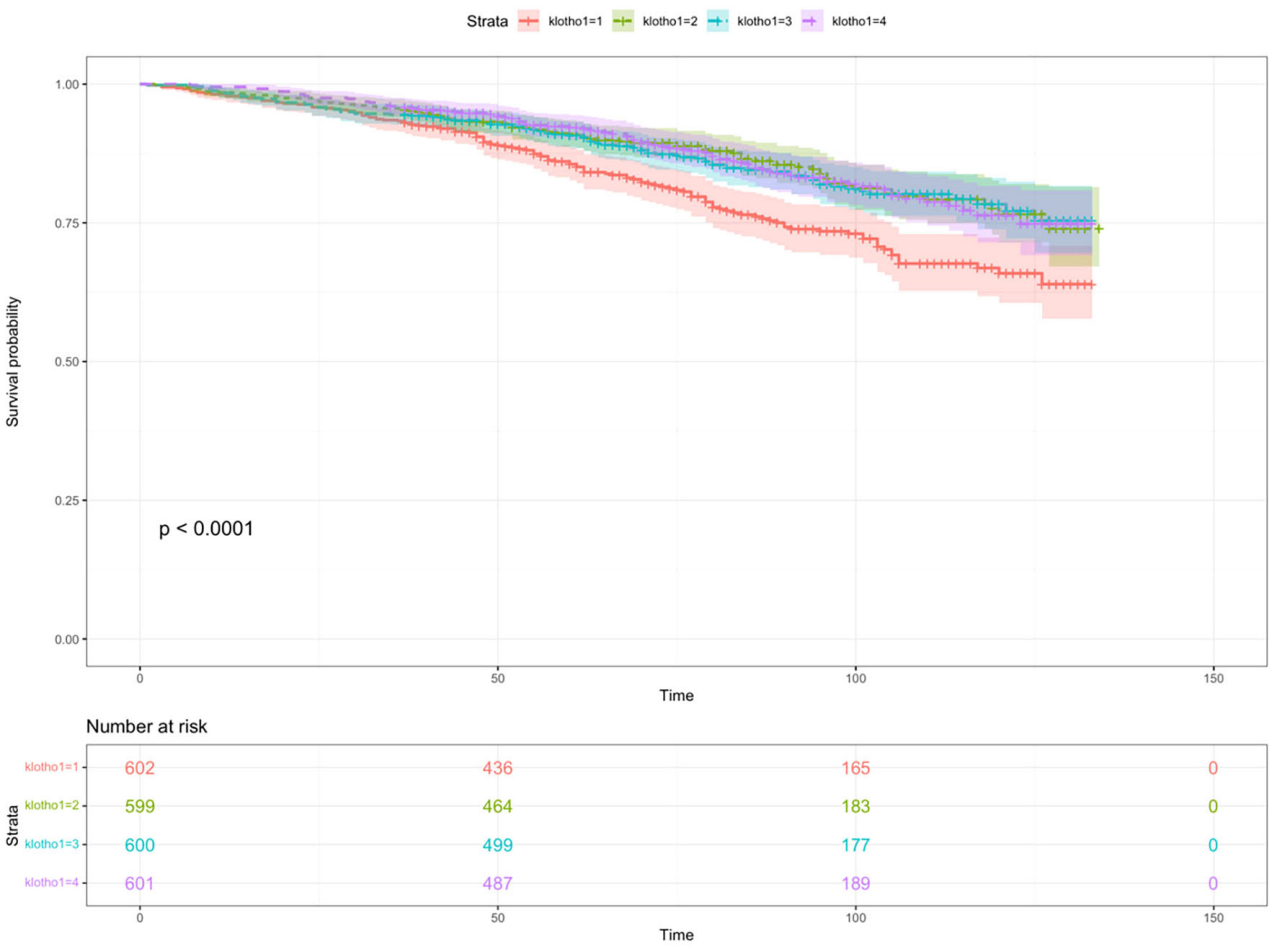
The Kaplan-Meier survival curve describing the relationship between klotho levels and all-cause mortality in patients with metabolic syndrome.

Additionally, the RCS analysis confirmed a significant non-linear relationship between serum klotho levels and all-cause mortality risk in patients with metabolic syndrome (P value for non-linearity <0.0001, total P value <0.0001). Kaplan-Meier survival curves were also plotted to demonstrate the all-cause mortality rates for the four quartiles of log2-transformed serum klotho, showing statistically significant differences (p<0.001) as shown in **Figure 6**. These findings support the notion that serum klotho levels play a significant role in the overall mortality risk among patients with metabolic syndrome.

### 3.4 Sensitivity analysis

As shown in **Table 2**, in the sensitivity analysis, participants with a death time of less than 30 months were excluded, and the results did not show significant changes. Compared to the Q1 group, the overall risk of death in the Q2, Q3, and Q4 groups decreased by 46% (HR=0.54, 95% CI=0.39-0.75, P<0.001), 46% (HR=0.54, 95% CI=0.38-0.75, P=0.004), and 32% (HR=0.68, 95% CI=0.49-0.93, P=0.016), respectively. Sensitivity analysis results indicated that serum klotho levels, transformed by log2, remained significantly associated with a decrease in overall risk of death.

**Table 2:** The relationship between Klotho levels in patients with metabolic syndrome and all-cause mortality rate was assessed after excluding patients who died within 30 months of follow-up.

	HR^1^	95% CI^2^	*P*-value
Klotho			
Q1	Reference		
Q2	0.54	0.39 - 0.75	<0.001
Q3	0.54	0.38 - 0.75	<0.001
Q4	0.68	0.49 - 0.93	0.016

^1^HR, Hazard Ratios.

^2^CI, Confidence Intervals.

## 4 Discussion

In this large nationally representative cohort of U.S. adults, we found a graded inverse association between serum Klotho levels and the prevalence of metabolic syndrome, independent of potential confounders including demographics, socioeconomics, and lifestyle factors. Moreover, higher Klotho levels strongly predicted lower risks of all-cause mortality among individuals with metabolic syndrome. Our study provides compelling evidence supporting Klotho as a novel protective factor against metabolic syndrome and an independent prognostic predictor. The prospective cohort design and linkage to longitudinal mortality data add to the significance of the findings. The population-based sample enhances generalizability to diverse real-world settings. Extensive adjustment for possible confounders including age, gender, race/ethnicity, smoking, PIR, and education level also represent major strengths.

Metabolic syndrome represents a cluster of metabolic abnormalities and cardiovascular risk factors, with central obesity, hypertension, dyslipidemia, and hyperglycemia as its principal components (1). The prevalence of metabolic syndrome has increased dramatically worldwide over recent decades. It now affects approximately 20-30% of the adult population globally, and up to 50% among older adults (2, 4). Metabolic syndrome significantly elevates the risks of developing type 2 diabetes, cardiovascular disease, chronic kidney disease, nonalcoholic fatty liver disease, and other metabolic disorders (3, 5–8). Moreover, numerous studies have demonstrated a 1.5- to 2.0-fold increase in cardiovascular mortality and a 1.5-fold elevation in all-cause mortality associated with metabolic syndrome (3, 7, 9). Therefore, metabolic syndrome confers a substantial health and economic burden both at the individual and societal levels. Identifying novel biomarkers and therapeutic targets is imperative to curb this growing public health threat.

Klotho is a transmembrane protein predominantly expressed in renal tubular cells that functions as an aging suppressor (10). Animal studies have revealed that Klotho-deficient mice exhibit multiple accelerated aging phenotypes, including shortened lifespan, arteriosclerosis, skin atrophy, osteoporosis, and cognitive impairment (11, 13). In humans, klotho deficiency is involved in the pathogenesis of various aging-related disorders, including cardiovascular disease, stroke, and chronic kidney disease (12, 14, 17).

Emerging evidence suggests that Klotho plays a vital role in metabolic regulation. Klotho activates insulin and insulin-like growth factor-1 (IGF-1) signaling by increasing the phosphorylation and glycosylation of their receptors (18, 19). This enhances insulin and IGF-1 receptor affinity and sensitivity. Klotho-overexpressing mice exhibit increased insulin sensitivity and resistance to diet-induced obesity (20). Conversely, Klotho loss-of-function mutant mice display impaired glucose tolerance and insulin resistance (21). In humans, lower Klotho levels are associated with increased fasting glucose, insulin resistance, increased glycated hemoglobin, and diabetes prevalence (22–24).

Regarding lipid metabolism, Klotho suppresses intracellular cholesterol biosynthesis by inhibiting HMG-CoA reductase (25). Klotho overexpression reduces triglyceride accumulation in the liver and circulation (26). Moreover, Klotho preserves endothelial function and prevents atherosclerosis by inhibiting vascular inflammation and oxidative stress (27). The pleiotropic metabolic benefits make Klotho an attractive candidate biomarker and therapeutic target for metabolic syndrome.

Klotho likely ameliorates metabolic syndrome through diverse mechanisms. As mentioned, Klotho enhances insulin sensitivity and improves lipid metabolism via modulating insulin/IGF-1 and HMG-CoA reductase signaling (18, 19, 21, 25, 26). Furthermore, Klotho activates the TRPV5 calcium channel in kidney tubules to increase calcium reabsorption (28). Elevated calcium can induce pancreatic β-cell dysfunction and peripheral insulin resistance (29, 30). Therefore, Klotho effectively ameliorates metabolic syndrome by increasing serum calcium. Previous studies have suggested a close association between Klotho levels and mineral metabolism as well as phosphate balance (10, 17).We further investigated the relationship between Klotho levels and blood calcium and phosphate concentrations, but did not find any underlying relationship between them, which may be closely related to the specific distribution of the metabolic syndrome population. Additionally, Klotho lowers serum phosphate by suppressing tubular phosphate reabsorption (31). Hyperphosphatemia promotes vascular smooth muscle cell transformation into osteoblast-like cells that calcify the vessel wall (32). Aberrant vascular calcification leads to increased arterial stiffness, impaired vasodilation, hypertension, and greater cardiac afterload (33, 34). Klotho prevents ectopic soft tissue calcification to ameliorate metabolic syndrome complications. Further research is warranted to elucidate the precise molecular pathways involved.

Our findings add to the emerging evidence supporting Klotho as a key regulator of metabolic homeostasis and a potential therapeutic target for metabolic syndrome. Recombinant Klotho protein administration could be a novel treatment approach. In mouse models, exogenous Klotho ameliorates vascular calcification, renal fibrosis, and cardiac hypertrophy (35–37). Phase 2 trials of Klotho protein therapy in chronic kidney disease patients are ongoing, showing acceptable safety and bioactivity profiles thus far (38). Developing targeted Klotho-based therapies may provide innovative management strategies to stem the rising tide of metabolic syndrome worldwide.

Several evident limitations of this study need to be acknowledged. First, the cross-sectional design of the study limited causal inference between Klotho and metabolic syndrome. Furthermore, while the sample size from the NHNAES database is nationally representative, its relative smallness might limit its power to detect minor effect sizes. Therefore, it is necessary to establish longitudinal cohort studies of Klotho biomarkers in the future. Clarifying how Klotho protein interacts with the genome, microbiota, and other factors influencing metabolic syndrome will have substantial research implications.

## 5 Conclusion

In summary, our study reveals a robust inverse association between circulating Klotho levels and metabolic syndrome prevalence and mortality. The results highlight the potential role of Klotho as a novel biomarker for early risk stratification and prognostication, as well as a promising therapeutic target for metabolic syndrome prevention and treatment. Further research is warranted to advance the translational potential of harnessing Klotho biology against this major global public health threat.

## Data availability statement

The raw data supporting the conclusions of this article will be made available by the authors, without undue reservation.

## Ethics statement

The studies involving humans were approved by National Center for Health Statistics Research Ethics Review Board. The studies were conducted in accordance with the local legislation and institutional requirements. Written informed consent for participation in this study was provided by the participants’ legal guardians/next of kin.

## Author contributions

LY: Data curation, Formal analysis, Visualization, Writing – original draft. YC: Data curation, Formal analysis, Visualization, Writing – original draft. NC: Investigation, Methodology, Writing – original draft. YZ: Investigation, Methodology, Writing – original draft. XZ: Investigation, Methodology, Writing – original draft. WS: Conceptualization, Methodology, Supervision, Writing – review & editing. JL: Conceptualization, Methodology, Writing – review & editing. XL: Conceptualization, Methodology, Supervision, Writing – original draft, Writing – review & editing.
